# "All Sorts and Conditions" of Women

**Published:** 1889-02-16

**Authors:** 


### The Doctor's Pulpit.

### "ALL SORTS AND CONDITIONS" OF WOMEN.

### Are Old Maids Failures?

Success to some is the very breath of life, and failure is the miasma of death. The stronger the personality, the more resolutely will it do battle against adverse fortune, and the more persistently will it search for "fresh fields" if the ordinary pastures have proved barren and profitless. In weaker natures there is less both of power and will to fight, and more manifest poverty of resource if defeat has been experienced on the common battlefields of life. The smaller the country, the fewer are the opportunities of its citizens; and the narrower the education and the lot of any individual, the more limited are the possibilities of success or distinction. Women have a more circumscribed platform than men on which to show the powers and capacities with which Nature has endowed them. By prescription, by education, by habit, they are alike "cabin'd, cribb'd, confin'd" within a narrow range of opportunities. By common consent of past ages, the one great and almost sole opportunity of a woman was marriage. When it failed, everything failed; life itself was a failure. The stronger women of the present age have thrown off the bondage of that tradition. They will not admit that woman's sole opportunity is marriage, and that the failure or success of life must be measured by the marriage standard alone. This is emphatically an evidence of capacity, of courage, of nobility, and of enlargement of mind. The power to stand alone, to think alone, to act alone, to succeed alone, or to fail alone—these are marked evidences of advancement on the lines of upward development. They who fail to understand these things show no comprehension of the "signs of their own times."

But though marriage is no longer the only opportunity of women, it is still the most natural and the most desirable, other things being equal. The greater the mental capacity of a woman, the more robust her character, the more is it to be wished, for the sake of the commonwealth and of the race, that she may marry and become the mother of children. A suitable marriage will not make her mental powers feebler, but stronger; will not make her sympathies narrower, but broader; will not make her experience shallower, but deeper. It will add to her natural endowments and character nothing that is unworthy, but many things that will make her altogether nobler and greater than she could ever have been without such experience. All women, however, cannot marry, and many of those whose mental endowments are the highest, are found among the ranks of the unmarried. There are a good many reasons for this. Commonplace men, of whom the world largely consists, are a little afraid to ask such women to marry, and the women themselves more or less consciously imagine in their hearts an ideal husband, and are very unwilling to let their actual experience fall below the level of their hopes and imaginings. Women, it is admitted, are more ideal than men, and much slower in dethroning the idols of the imagination. Let neither man nor woman lament that, but rather be thankful for it.

It is not all loss when clever and high-minded women do not marry. Though the world ultimately be poorer, the immediate genera ion may be all the richer for it. The families and acquaintances of such women, though sometimes both unconscious and altogether unworthy of their gifts and endowments, are blessed and enriched by them in a thousand ways. The women themselves may be often discouraged by the commonplace blindness of their relatives and acquaintances, which refuses to admit the value of higher and worthier thoughts and aims; but the influence of high character and intelligence can by no possibility be wasted, even though it have to operate upon mere aimless dullards. Besides, many of the women we are speaking of have pushed their way through the thorns and briars of family and social difficulties, and by pen or pencil, or other worthy method, have made themselves powers in the land. Though no man of sufficient nobility came to gather these flowers in their early sweetness and bloom, yet has their quality been so rare that it has compelled attention from a busy, but not entirely stupid and vulgar world. The work that they do is of a kind which men could probably never do, and which perhaps even they themselves might hardly find time for if the cares of family life were theirs. But in spite of adverse opinion, we hold that a married woman of undoubted mental gifts would be well advised to employ capable aid in domestic affairs, and to devote herself to the higher work to which nature has pointed her. A man with a family is not the less fitted to be a man of letters, or a statesman, or a great preacher, because of his children; rather, perhaps, he is all the more competent. Why should not a woman with a family devolve much of the care of her house on intelligent subordinates, and give up her best energies to the highest intellectual occupation for which she has proved her fitness?

But our present business is with unmarried women who have been passed by in the marriage race—flowers which no man hath gathered. The highest and the best of them do not mind their lot in the least. Many are, indeed, unmarried from choice. Some who are still in spinsterhood could have been married ten times over. It is to be feared, however, that not a few are both soured and injured by the neglect or unintelligent dulness of the other sex. Men choose women, not women men. Men have the initiative. It is for them to take the first step. Readily as we may admit in the abstract that women ought to have equal rights with men at such an important crisis of their lives, neither men nor women can put away the "feeling" that it is for a man to make advances. It is quite easy to conceive of circumstances in which a man, however deeply he might wish, could by no possibility bring himself to make the smallest forward movement. It is as easy to imagine cases in which a word or a look from a woman might save the happiness of two noble lives from complete and final shipwreck. What should be done in such a case? No general rule can be laid down. Speaking as a man, the present writer admits that he himself would not honour less, but more, a woman who, finding no other way of solving a difficult problem, should frankly approach the initiative. *Honi soit qui mal y pense.* A noble woman need not fear to trust herself. Such is the fixed and immovable grandeur of soul of the best women that they cannot practically do wrong. Providence has endowed them with souls in which unworthy motives can never find lodgment or even access.

Granted, however, all possible freedom which a high-minded woman could desire, there will still remain a considerable number of women who have not married, and who consider themselves more or less of failures in consequence. Is this decision of theirs on the subject the one which commends itself to the informed judgment of the present age? It certainly is not. The failure to marry should no more be considered a life-failure than the want of wealth or of high birth. A woman of native capacity and force of character will simply look upon the marriage disappointment as the closing of one of many possible avenues which might have led to prosperity and happiness. She will consider what other field may offer scope for her care and peculiar powers. Thanks to the spirit of the times, but thanks most of all to the courage and determination of women themselves, all sorts of closed preserves have been opened to them, and they, like men, have almost "all the world before them where to choose." It is for them to choose resolutely, and if possible also wisely; but at any rate, resolutely. Neither man nor woman can always be sure that the best possible course has been chosen at any given crisis; but both can be sure of this, that whatever right course it is decided to follow shall be followed with all the heart and mind and strength. To a capable woman, as to a capable man, a dozen doors of work may be opened, and it may be quite impossible at the moment to decide which of the dozen it is best to enter. But the choice having been once made, there is no longer room for indecision or regret, but only for using every possible effort to bring the work selected to a prosperous issue. The greatest happiness of woman, as of man, is usually found in the trusted companionship and mutual help of the married state; but failing that, there is for both sexes a full measure of wholesome content and prosperity in self-reliant work and a Wellingtonian victory achieved over all obstacles by courage and unflagging resolution. Happily for women, as for men, the work which may at first seem a hard and disagreeable necessity generally results in improved health, increased capacity, and a buoyant hopefulness which is the presage and impetus of final and assured success.

*(To be continued.)*

